# PROX1 interaction with α-SMA-rich cancer-associated fibroblasts facilitates colorectal cancer progression and correlates with poor clinical outcomes and therapeutic resistance

**DOI:** 10.18632/aging.205447

**Published:** 2024-01-18

**Authors:** Shiue-Wei Lai, Yi-Chiao Cheng, Kee-Thai Kiu, Min-Hsuan Yen, Ying-Wei Chen, Vijesh Kumar Yadav, Chi-Tai Yeh, Kuang-Tai Kuo, Tung-Cheng Chang

**Affiliations:** 1 Department of Internal Medicine, Division of Hematology and Oncology, Tri-service General Hospital, National Defense Medical Center, Taipei, Taiwan; 2 Department of Surgery, Division of Colon and Rectal Surgery, Tri-Service General Hospital, National Defense Medical Center, Taipei, Taiwan; 3 Department of Surgery, Division of Colorectal Surgery, Taipei Medical University Shuang-Ho Hospital, Taipei, Taiwan; 4 Department of Surgery, School of Medicine, College of Medicine, Taipei Medical University, Taipei 110, Taiwan; 5 Department of Internal Medicine, Division of Gastroenterology and Hepatology, Shuang-Ho Hospital, New Taipei City, Taiwan; 6 Department of Medical Research and Education, Taipei Medical University Shuang-Ho Hospital, New Taipei City 23561, Taiwan; 7 Continuing Education Program of Food Biotechnology Applications, College of Science and Engineering, National Taitung University, Taitung 95092, Taiwan; 8 Department of Surgery, Division of Thoracic Surgery, School of Medicine, College of Medicine, Taipei Medical University, Taipei 110, Taiwan; 9 Department of Surgery, Division of Thoracic Surgery, Taipei Medical University Shuang-Ho Hospital, New Taipei City 23561, Taiwan

**Keywords:** colorectal cancer, prognosis, biomarker, PROX1, α-SMA, cancer-associated fibroblast, metastasis

## Abstract

Background: The tumor microenvironment (TME) plays a vital role in tumor progression through intricate molecular interactions. Cancer-associated fibroblasts (CAFs), notably those expressing alpha-smooth muscle actin (α-SMA) or myofibroblasts, are instrumental in this context and correlate with unfavorable outcomes in colorectal cancer (CRC). While several transcription factors influence TME, the exact regulator causing CAF dysregulation in CRC remains elusive. Prospero Homeobox 1 (PROX1) stands out, as its inhibition reduces α-SMA-rich CAF activity. However, the therapeutic role of PROX1 is debated due to inconsistent study findings.

Methods: Using the ULCAN portal, we noted an elevated PROX1 level in advanced colon adenocarcinoma, linking to a poor prognosis. Assays determined the impact of PROX1 overexpression on CRC cell properties, while co-culture experiments spotlighted the PROX1-CAF relationship. Molecular expressions were validated by qRT-PCR and Western blots, with *in vivo* studies further solidifying the observations.

Results: Our study emphasized the connection between PROX1 and α-SMA in CAFs. Elevated PROX1 in CRC samples correlated with increased α-SMA in tumors. PROX1 modulation influenced the behavior of specific CRC cells, with its overexpression fostering invasiveness. Kaplan-Meier evaluations demonstrated a link between PROX1 or α-SMA and survival outcomes. Consequently, PROX1, alone or with α-SMA, emerges as a CRC prognostic marker. Co-culture and animal experiments further highlighted this relationship.

Conclusion: PROX1 appears crucial in modulating CRC behavior and therapeutic resistance within the TME by influencing CAFs, signifying the combined PROX1/α-SMA gene as a potential CRC prognostic marker. The concept of developing inhibitors targeting this gene set emerges as a prospective therapeutic strategy. However, this study is bound by limitations, including potential challenges in clinical translation, a focused exploration on PROX1/α-SMA potentially overlooking other significant molecular contributors, and the preliminary nature of the inhibitor development proposition.

## INTRODUCTION

Colorectal cancer (CRC) with a high prevalence is one of the major causes of cancer-linked mortality around the world [1]. Due, to the high rate of local and distant metastasis and disease relapse, it correlates to a poor prognosis [2]. The 5-year survival rate of metastatic CRC patients is just 10–20% as compared to 90% for patients with a localized CRC [3]. The resistance to current therapy is a major obstacle against CRC treatment, which is often because of the presence of cancer stem cells (CSCs), a subgroup of primary CRC cells that can self-renew, and is the leading cause of chemoresistance and recurrence [4, 5]. There is extensive research relating to metastasis, relapse, and disease-associated mortality in patients with CRC that has been studied and reported, but underlying mechanisms still remain vague. The current understanding emphasizes the importance of the tumor microenvironment (TME) in cancer development. The TME, primarily comprising the immediate vicinity around tumor tissues, plays a crucial role in tumorigenesis. Alterations in the TME are closely associated with the onset and development of cancer. Within the TME, the tumor stroma is a key component that influences various aspects of cancer biology. It is implicated in initiating and advancing cancer, contributing to the spread of the disease, and impacting the effectiveness of cancer treatments [4, 5].

The tumor stroma covers lymphatic networks and a grid of fibroblast-generated extracellular matrix (ECM), monocytes, macrophages, and different inflammatory cells [6]. Intermolecular interactions within this active TME alter tumor fate, and CSC biological activity plays a crucial role in tumor progression [7]. Notably α-smooth muscle actin (α-SMA)-rich cancer-associated fibroblasts (CAFs) govern the various stroma biological processes [8]. Following, α-SMA-expressing CAFs demonstrate a different gene expression pattern to normal fibroblasts and may confer CSC/oncogenic phenotypes on normal epithelial cells [9–11]. They enhance chemoresistance and cancer progression [12, 13] through multiple processes, such as compositing and remodelling of the ECM, as well as secretion of several soluble factors that likely can interact with the cancer cells, and disruption of immunity [14–16]. The inhibition of CAFs can be a promising treatment strategy for cancer [17, 18], yet many challenges have arisen in its clinical practice.

A critical issue is the limited knowledge regarding the varied characteristics of different CAF populations in the TME. An impactful, yet unexplored approach to address this challenge is to identify and understand the principal transcriptional regulators controlling each CAF subgroup. These core transcriptional regulators include the NANOG, OCT4, and SOX2 which regulate gene expression and determine cell fate and differentiation, for example in regulating the pluripotency of stem cells [19]. However, no key molecular gene transcription factors have been reported that specifically alter the CAF’s molecular-regulatory circuits. Therefore, finding the molecular transcriptional factors in CAFs modulation may help in the precise classification of various CAF subsets, than that for already known classical markers such as FAP, SMA, and FSP1 [20].

It has also been observed that in CRC, the dysregulated Wnt pathways influence the transcription factor Prospero homeobox 1 (PROX1).[21], which is overexpressed in multiple cancers. Altered PROX1 expression is also reported involved in regulating the fate of stem cells and progenitor cells [22]. In the healthy intestine, PROX1 is present in only a limited number of secreting cells. [23]. Altering PROX1 expression in colorectal cancer (CRC) cells, which exhibit characteristics similar to stem cells, can impact tumor size and the population of stem cells. Specifically, decreasing PROX1 expression is associated with a reduction in both tumor size and the number of stem cells [21]. It has also been reported to suppress brain tumors by preventing neuroblast self-renewal [24, 25]. However, PROX1-targeted therapies for CRC have not yet been developed for clinical therapies, and understanding its role in other CRC phenotype regulations, particularly in the CRC-TME, prompted us to commence the current study.

The present study involved an extensive bioinformatic analysis using publicly accessible data from The Cancer Genome Atlas Colon Adenocarcinoma (TCGA-COAD) via the ULCAN portal. This was complemented by *in vitro* investigations of PROX1 in colorectal cancer (CRC), focusing on examining the effects of its loss or gain of function. The methodologies included immunoblotting, real-time quantitative polymerase chain reaction (RT-qPCR), immunohistochemical (IHC) staining, and various functional assays, to assess the role of PROX1 together with α-SMA expression within CRC cell lines and patient tissues. Additionally, interactions and correlations between PROX1 and α-SMA expression were explored concerning disease progression and clinical outcomes, aiming to substantiate the role of PROX1 in CAFs, particularly in CRC-TME modulation. Notably, PROX1 and α-SMA expressions were prominently elevated in the tissues of CRC patients. Furthermore, suppressing PROX1 reduced the processes of cell proliferation, migration, and invasion in both *in vivo* and *in vitro* environments, while increasing PROX1 expression had the opposite effect, enhancing these cellular activities. Flow cytometry illustrated that reduced PROX1 expression resulted in cell G1 phase arrest, and its overexpression facilitated the G1–S phase transition. Through IHC and immunofluorescence analyses, significant expressions of PROX1 and α-SMA were uncovered within the cancer cells’ tumor matrix and a strong correlation was observed between PROX1 and α-SMA expression in CRC.

The study revealed that high levels of PROX1 and α-SMA in CRC tissues are linked to increased tumor cell invasiveness and higher tumour-node-metastasis (TNM) stages. Furthermore, co-culture experiments demonstrated a significant link between PROX1 expression and the emergence of a CAF-like phenotype and markers, potentially leading to chemoresistance and CSC phenotype. Though the regulatory influence of PROX1 on cancer progression and chemoresistance is increasingly recognized, the fundamental mechanisms are yet to be clarified.

## MATERIALS AND METHODS

### Bioinformatics analysis

The expression of PROX1 and associated genes in the TCGA database was analyzed using the UALCAN online portal (http://ualcan.path.uab.edu/). Detailed steps for how to use this portal were described earlier [26]. Expression and association between genes in the CRC cell line and finalization for further study were based on DepMap online analysis (https://depmap.org/portal/).

### Patients and samples

The 164 patients underwent primary CRC resection in the Department of Surgery between August 2009 and December 2011 at Taipei Medical University Shuang-Ho Hospital. Of them, we excluded patients with microscopically or grossly noncurative resection, inadequate lymph node dissection (fewer than 12 lymph node dissections), ulcerative colitis, synchronous malignancies, and less than 30 days of pre-mortality hospitalization as well as those who underwent neoadjuvant therapy. In the final analysis, we incorporated a total of 164 patients with colon adenocarcinoma who underwent curative surgical resection have been included in this study. Surgically excised specimens of cancerous and paired normal tissue were obtained from the patients for IHC staining, Western blot and qRT-PCR analysis. Data on patient age, sex, tumor size and depth, lymph node invasion and metastasis, vascular invasion, distant metastasis, clinical stage, and histological grade were obtained from clinical and pathological records (Table 1). Relapse was defined as new lesions that were discovered using imaging modalities such as radiography, ultrasonography, and computed tomography. All patients had followed up for survival analysis. The median follow-up period was 16.9 months (range: 2.7–36.7 months). The study protocol has been approved by the Ethical Committee of Taipei Medical University Shuang-Ho Hospital, Taipei, Taiwan. Clinical samples were collected from Taipei Medical University Shuang-Ho Hospital. All enrolled patients provided written informed consent for their tissues to be used for scientific research. This study received approval from the Institutional Review Board of Taipei Medical University Shuang-Ho Hospital in Taipei, Taiwan. It was conducted under the guidelines of the Declaration of Helsinki for biomedical research and adhered to the standard institutional protocols for human research at Taipei Medical University Shuang-Ho Hospital, Taipei, Taiwan (Certificate of TMU-JIRB Approval N202303130).

**Table 1:** Correlation between PROX1 or α-SMA expression and patients’ clinicopathological characteristics.

**Clinical characteristics**	**PROX1 expression**		***P*-value**	**α-SMA expression**		***P*-value**
	**High (*n* = 116)**	**Low (*n* = 48)**		**High (*n* = 130)**	**Low (*n* = 34)**	
Gender			0.70			0.60
Male	57	22		64	15	
Female	59	26		66	19	
Mean age	66.0	62.9	0.16	65.4	63.9	0.079
Location			0.29			0.70
Right side	44	14		45	13	
Left side	72	34		85	21	
Size			**<0.001**			**<0.001**
≤5 cm	44	41		55	30	
>5 cm	72	7		75	4	
Histology type			**<0.001**			**<0.001**
Well	0	10		0	10	
Moderate	100	35		114	21	
High or mucinous	16	3		16	3	
Serosal invasion			**<0.001**			**<0.001**
Positive	111	20		123	8	
Negative	5	28		7	26	
Lymphatic invasion			**0.002**			**0.004**
Positive	65	14		70	9	
Negative	51	34		60	25	
Venous invasion			0.089			0.11
Positive	53	15		58	10	
Negative	63	33		62	24	
Lymph node metastasis			**<0.001**			**<0.001**
Positive	69	9		76	2	
Negative	47	39		54	32	
Distant metastasis			**0.016**			0.055
Positive	13	0		13	0	
Negative	103	48		117	34	
Stage						
I	0	27	**<0.001**	2	25	**<0.001**
II	45	12		50	7	
III	58	9		65	2	
IV	13	0		13	0	

### Cell culture

All the human CRC cell lines such as HT-29 (HTB-38, ATCC), HCT116 (CCL-247, ATCC), SW480 (CCL-228, ATCC), SW620 (CCL-227, ATCC), DLD-1 (CCL-221, ATCC), and Caco-2 (HTB-37, ATCC) used in our study were cultivated at 37°C in humidified 5% CO_2_ incubator with RPMI-1640 medium supplemented with 10% fetal bovine serum, 100 U/mL penicillin, 100 μg/mL streptomycin, and 20 mM L-glutamine. Further, the cell was passaged on confluency and the medium was changed every 72 h (Approval BSL-2-0192).

### Cell counting kit-8 cell viability assay

A cell counting kit-8 (CCK-8) assay (Dojindo Laboratories, Kumamoto, Japan) was used to evaluate CRC cell survival and proliferation as per the producer’s protocol.

### CRC cells cocultured with CAFs

The co-culture protocol was modified from the method initially described by Sung et al. [27]. Initially, 8 × 10^4^ CAFs were plated in a 10-cm dish. On a subsequent day, cancer cells (SW-480, SW-620, HCT116, and HT-29 cells were added at a density of 4,000 cells per 10-cm petri dish. The ratio of CAFs to cancer cells varied between 50:1 and 10:1, contingent on the specific cell type utilized. Following an incubation period, the spent culture medium was substituted with a fresh one. Twenty-four hours after this replacement, the cells were subjected to treatment with a conditioned medium or left untreated, preceding the execution of subsequent experiments.

### PROX1 knockdown and overexpression study

Mammalian PROX1 lentivirus containing short hairpin (sh) PROX1 RNA and an empty shRNA vector were purchased from Thermo Fisher Scientific (USA) and prepared under strict adherence to the manufacturer’s instructions. Two clones of shRNA were used to effectively knock down PROX1 expression: PROX1 shRNA#1 and shRNA #2. In addition, the full-length human PROX1 cDNA was inserted into the pcDNA3.1 vector to generate an overexpression vector pcDNA-PROX1 construct. Cells were transfection plasmids using Lipofectamine 2000 (Invitrogen; Thermo Fisher Scientific) to induce overexpression.

### Western blotting and RT-qPCR

Protein expression was analyzed through Western blotting. After all the respective experiments, whole-cell lysates were prepared using RIPA lysis buffer, and cell protein lysates were isolated using a protein extraction kit (QIAGEN, USA) and quantified using the Bradford Protein Assay Kit (Beyotime, USA). The prepared cell lysates were subjected to sodium dodecyl sulfate–polyacrylamide gel electrophoresis and then transferred to a polyvinylidene fluoride membrane. Membranes were probed with specific antibodies at 4°C overnight and then underwent secondary antibody incubation (room temperature, 1 h). Furthermore, the RT-qPCR was performed by isolating total RNA using a TRIzol-based protocol (Life Technologies, USA) following the manufacturer’s instructions. Briefly, 200 ng of total RNA was reverse-transcribed using a OneStep RT-PCR Kit (QIAGEN, Taiwan), and PCR was performed using a Rotor-Gene SYBR Green PCR Kit (400, QIAGEN, Taiwan). Antibody details, along with the dilution and gene-specific primers used in this study were described.

### Cell invasion and migration assays

Vertical cell motility was assessed using Matrigel invasion assay in a Boyden chamber, as described by Ha et al., and horizontal migration was assessed using the wound-healing assay, previously described [28].

### Colony-formation assay

The colony-formation assay was carried out using a previously explained protocol [29] with adjustments. In short, 500 CRC cells (suppressed or overexpressed PROX1) were sown in six-well plates. The cells were permitted to grow for 1 week and then harvested, fixed, and counted to show the effect of PROX1 loss or gain of function on the CRC cells’ self-renewal properties.

### Flow cytometric analysis

After the stable shRNA transfection and overexpression experiments, along with all the respective controls (vector only), the transfected CRC cells were seeded into six-well plates, cultured in RPMI-1640 medium added 10% fetal bovine serum, and incubated at 37°C in 5% CO_2_ for 24 h. Cell apoptosis was assayed using a PE Annexin V Apoptosis Detection Kit I (BD Biosciences, USA) following the manufacturer’s instructions. Cells were trypsinized with 0.25% trypsin–ethylenediaminetetraacetic acid solution washed twice with cold phosphate-buffered saline and stained with Annexin V-PE (5 μL) and 7-AAD (5 μL) in binding buffer. After incubation at room temperature for 15 min, cell apoptosis was analyzed using a BD fluorescence-activated cell sorting Aria III flow cytometer.

### Immunofluorescence analysis

The human colon cancer cells (SW620 and HCT116), after all, experiments, were plated in six-well chamber slides for 24 h for immunofluorescence analysis. The cells were fixed with 2% paraformaldehyde and probed with primary antibodies against fibronectin. A fluorophore-conjugated secondary antibody was added to evaluate positive signals captured using a Zeiss Axiophot (Carl Zeiss, Jena, Germany) fluorescence microscope. The nuclei of viable cells were detected using DAPI staining.

### IHC analysis

PROX1 and α-SMA expressions in primary tumors were quantified using IHC analysis. All IHC staining was scored independently by two separate pathologists. Slides were examined under a low-resolution (40×) microscope to identify tissue regions with the highest burden of PROX1- or α-SMA-positive cells. Ten fields of tumor nests were selected, and expression was evaluated in 1000 tumor cells (100 cells per field) under high resolution (400×). Tumor cells stained with PROX1 or α-SMA antibody were defined as positive. IHC scoring was performed using the immunoreactivity scoring system (IRS). Category A documented the intensity of immunostaining as 0 (no immunostaining), 1 (weak), 2 (moderate), or 3 (strong). Category B documented the percentage of immunoreactive cells as 0 (negative), 1 (scattered positive cells: 1%), 2 (2–10% positive cells), 3 (11–50%), 4 (51–80%), or 5 (>80%). The summation of categories A and B resulted in an IRS score ranging from 0 to 8 for each case, with scores of 0–4 and 5–8 indicating low and high expression, respectively.

### *In vivo* studies

The study involving animals was sanctioned by the Animal Care and Use Committee at Taipei Medical University Shuang-Ho Hospital, Taipei, Taiwan (Approval Protocol # Taiwan Medical University-LAC2022-0472). We utilized 5-week-old female BALB/c athymic nude mice, each weighing approximately 20 grams, sourced from BioLASCO, Taiwan. These mice were kept in a sterile environment with access to sterilized food and water.

Initially, each mouse received a subcutaneous injection near the right hind leg with 1 × 10^6^ HCT116-PROX1-shRNA#1 cell. Once palpable tumors developed (around a volume of ~100 mm^3^), the total 10 mice were categorized into two groups: the control group (with shRNA-empty vector; *n* = 5) and the PROX1-shRNA#1 group (*n* = 5). Tumor growth was monitored weekly, with the volume estimated using the formula: 1/2 (length × width^2^). Post four weeks, the mice were euthanized humanely using a 5% isoflurane vaporizer, a standard euthanasia method for small animals at the Laboratory Animal Center (LAC), Taipei Medical University. Subsequently, tumors were extracted and prepared for further analysis.

### Statistical analysis

To assess the differential expression of PROX1 between cancerous tissues and their corresponding normal colonic tissues, we employed a paired *t*-test. This test allowed us to determine if there were significant differences in PROX1 levels between these two tissue types. For a more comprehensive analysis of PROX1 and α-SMA expression, we utilized two primary statistical tests. The student’s *t*-test was employed when comparing the means of two independent groups. In situations where multiple groups were involved, we opted for the one-way analysis of variance (ANOVA) followed by post hoc tests. These post hoc tests helped in identifying specific group differences after the ANOVA indicated significant variances among the groups. Furthermore, to explore the relationship between PROX1 and α-SMA expression and various clinicopathological parameters, unpaired *t*-tests were utilized. This approach helped in determining if the expression levels of these genes had any significant associations with specific clinical or pathological characteristics of the samples. Throughout our analysis, we adhered to a significance threshold of *p* < 0.05. All the data were repeated thrice, and any result with a *p*-value below this threshold was considered to indicate a statistically significant difference or association.

### Availability of data and materials

The data sets used and analysed in the current study are available from the corresponding author in response to reasonable requests.

## RESULTS

### PROX1 and α-SMA overexpressed in CRC patient samples and cell lines

**Figure 1:**
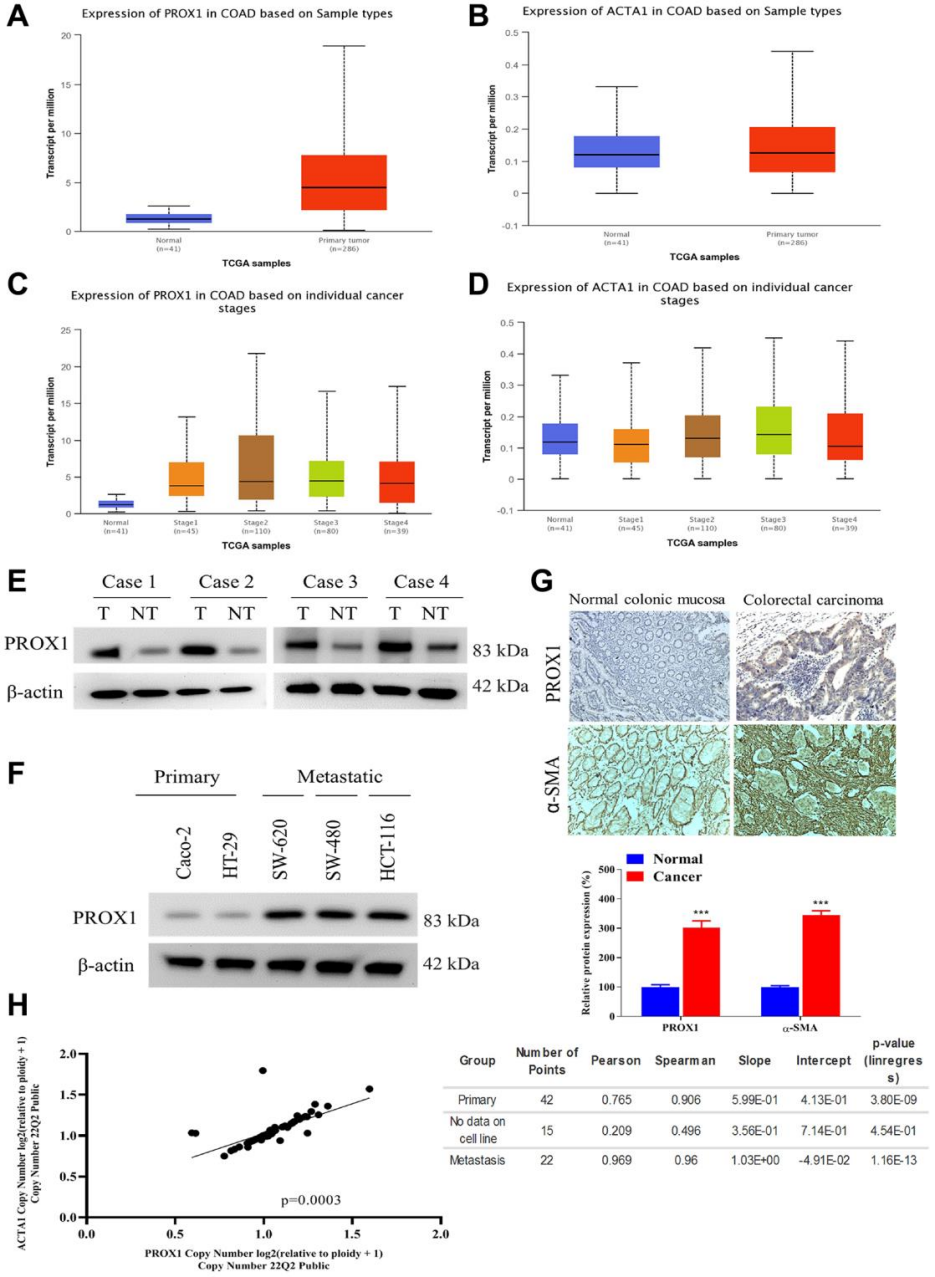
**PROX1 and α-SMA overexpressed in CRC clinical samples and cell lines.** (**A**, **B**) TCGA COAD dataset available from UALCAN shows, that PROX1 and α-SMA are upregulated in colorectal adenocarcinoma tissues. (**C**, **D**) PROX1 and α-SMA expression in COAD cases on the indivisible cancer stage from TCGA COAD dataset available from UALCAN. Western blot analysis of PROX1 expression in (**E**) paired tumor and non-tumor colorectal clinical samples and (**F**) human colorectal cancer cell lines, Caco-2, HT-29, SW-480, SW-620 and HCT116. β-actin is used as loading control; (**G**) Immunohistochemical analysis of PROX1 or α-SMA expression in normal colonic and colorectal tumor samples. (**H**) DepMap analysis of correlation expression of PROX1 and α-SMA.

The TCGA-COAD-RNA-sequencing was visualised using the UALCAN online portal (http://ualcan.path.uab.edu/). Which demonstrated the mean expression of PROX1 and α-SMA were significantly higher in COAD patients compared to adjacent normal tissue (*p* < 0.001; Figure 1A–1D). Furthermore, Western blotting and IHC analysis demonstrated, PROX1 expression was significantly higher in the human colon adenocarcinoma samples than in its corresponding non-tumor counterparts (Figure 1E, 1F). Importantly, PROX1 was localized, similar to α-SMA, mainly in the cytosolic and membranous portions of the cancer cells (Figure 1G). To choose cell lines for further experiments, the co-expression of PROX1 and α-SMA (ACTA1) in various CRC cells was analyzed using the DepMap database, accessed on October 30, 2022 (Figure 1H). The most significant PROX1 expression was observed in the metastatic CRC cell lines HCT116, SW-620, and SW-480, followed by HT-29 and Caco-2 cells, which are derived from primary CRCs. The following results affirmed the hypothesis that PROX1 facilitates CRC metastasis and progression and proved a strong correlation between PROX1 and α-SMA expression.

### PROX1 expression regulates the CRC malignant phenotype

**Figure 2:**
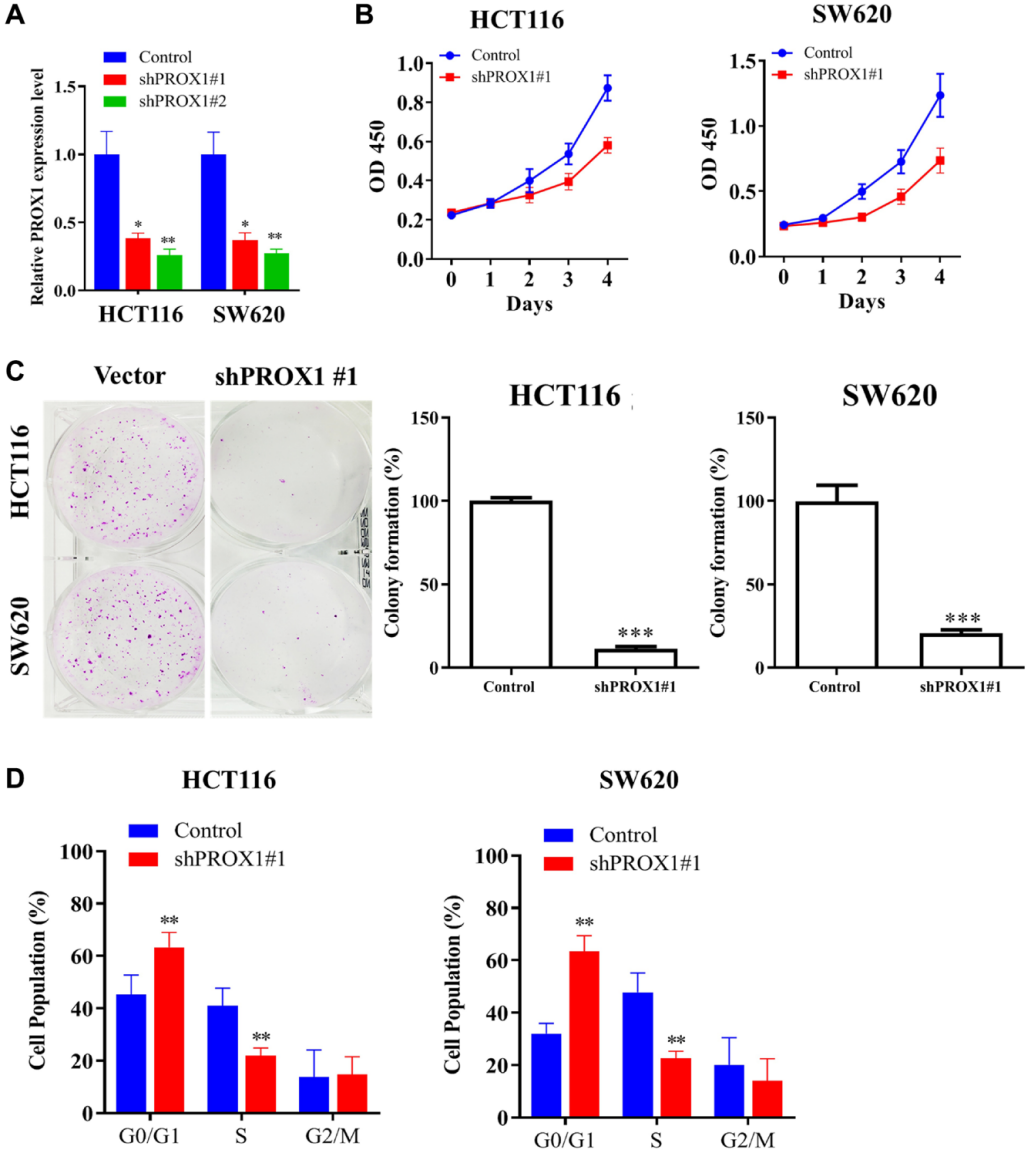
**Effect of PROX1 knockdown on CRC malignant phenotype.** (**A**) The mRNA expression level of PROX1 after shRNA transfection was evaluated by RT-PCR. (**B**) CCK-8 assay of sh-NC and PROX1 shRNA #1- transfected in HCT116 and SW 620 cells. (**C**) Colony formation assay analysis for the inhibitory effect of PROX1 knockdown on HCT116 and SW 620 cell migration. (**D**) Flow cytometry analysis of cell cycle phases distribution of HCT116 and SW 620 cells following the PROX1-shRNA infection. The experiments were performed thrice, and the data are presented as the mean ± standard deviation. ^*^P < 0.05 and ^**^P < 0.01 vs. sh-NC.

The expression of PROX1 was seen upregulated in CRC clinical samples and cell lines, with the highest expression observed in HCT116 and SW620 cells; therefore, these cell lines have been selected for further experiments. Exploring the role of PROX1 in CRC cells, empty shRNA vector and PROX1-shRNA#1 and #2 lentiviral constructs were transfected into HCT116 and SW620 cells. PROX1 knockdown was confirmed through RT-qPCR analysis (Figure 2A; the relative expression is shown in the bar plot). Additionally, cell viability assays (CCK-8) and colony formation assays demonstrated that PROX1 inhibition in CRC cells significantly decreased the viability and colony-forming abilities of the cells (*p* < 0.01; Figure 2B, 2C). To identify the pro-proliferative role of PROX1, a flow cytometric assay of the cell cycle was performed; PROX1 knockdown results in significant G1 phase arrest (*p* < 0.01; Figure 2D) of CRC cells.

### PROX1 inhibition reduces the invasiveness of SW620 and HCT116 cells

**Figure 3:**
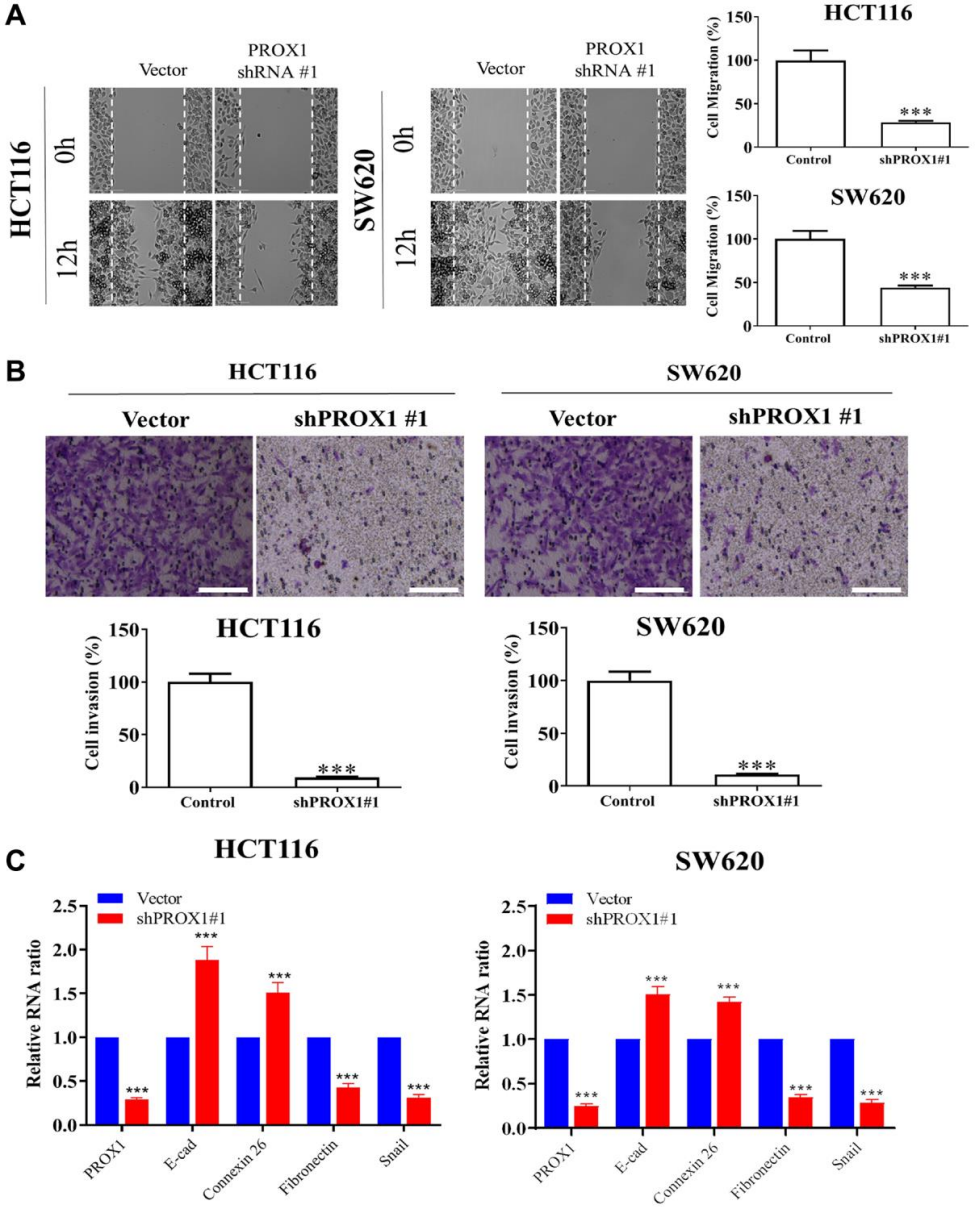
**PROX1 knockdown reduces the migration and invasiveness of SW620 and HCT116 cells.** (**A**) Wound healing assay for the inhibitory effect of PROX1 knockdown on HCT116 and SW620 cell migration. Cell migration into the wounded area was quantified based on the dashed line as time 0. Images were taken immediately after scratching, 0 h and 16 h later. Original magnification, ×200. Transwell invasion assay shows the invasive ability of (**B**) HCT116 and SW-620, significantly suppressed in PROX1 shRNA-infected cells. (**C**) RT-PCR shows PROX1 knockdown suppressed transcript expression of Fibronectin and Snail while upregulating E-cadherin and Connexin 26. GAPDH is used as an internal control. Data are presented as mean ± SEM. ^*^p < 0.05, ^**^p < 0.01, ^***^P < 0.001.

Migration assays and transwell invasion assays of CRC cells revealed, shRNA-PROX1 mediated inhibition of PROX1 significantly decreased HCT116 and SW620 cell migration (Figure 3A) and invasion when compared with transfection with the empty shRNA vector (*p* < 0.01; Figure 3B). The epithelial–mesenchymal transition (EMT) may be involved in cancer metastasis [30]. Therefore, to investigate the potential mechanism of CRC cell metastasis, we examined EMT marker expression in CRC cell lines by performing qRT-PCR. As presented in Figure 3C, PROX1-shRNA#1 knockdown, downregulated the EMT in CRC cells, significantly reduced the expression of fibronectin and mesenchymal marker Snail transcripts and upregulated the epithelial markers; E-cadherin and connexin 26. To investigate how PROX1 overexpression promoted CSCs properties in colonic neoplasms, we examined the PROX1 regulatory pathway. First, the expression of the PROX1 pathway downstream target genes and CAF markers were examined, and the results demonstrated that PROX1 promoted cancer progression, following the results of the migration and invasion assays. The motility and invasiveness of the CRC cell were significantly attenuated compared with those of the cells transfected with the control vector.

### PROX1 overexpression promotes CRC proliferative, migratory and invasion phenotype *in vitro*

**Figure 4:**
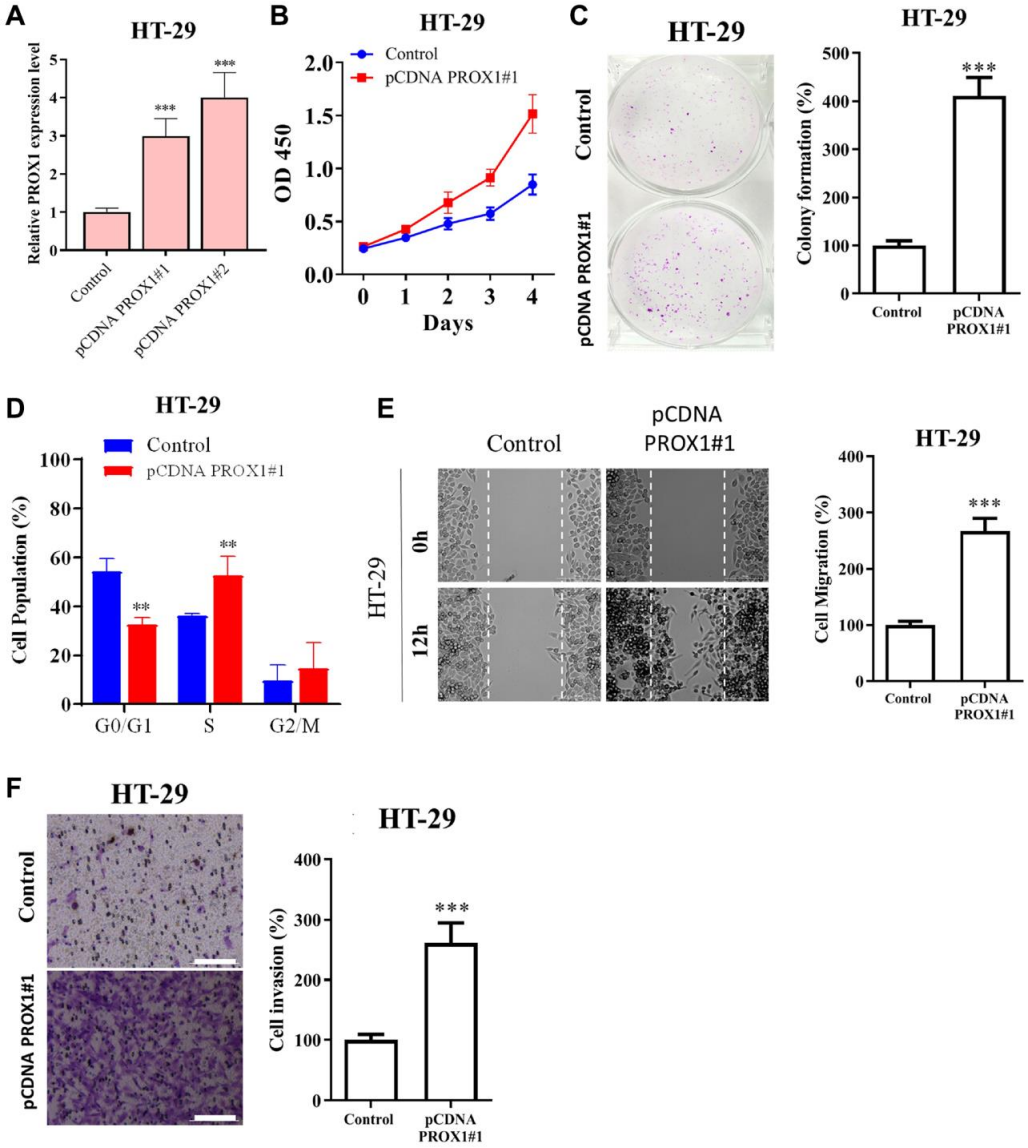
**PROX1 overexpression promotes the growth, migration, and invasion of colon adenocarcinoma cells.** HT-29 cells were transfected with either the pcDNA-PROX1 overexpressing vector or empty vector control. (**A**) RT-qPCR and (**B**) proliferation and (**C**) colony formation capability were estimated by CCK-8 and colony formation assays, respectively. (**D**) Flow cytometry showed the distribution of pcDNA-PROX1-transfected HT-29 cells in the G1, S, and G2/M phases of the cell cycle. (**E**) Cell migration ability was assessed by wound healing and (**F**) Invasion was assessed by the Transwell invasion assay in pcDNA-PROX1-transfected HT-29 cells and control cells. Magnification, ×100. Scale bar, 100 μm. The experiments were performed three times, and the data are presented as the mean ± standard deviation. ^*^P < 0.05 and ^**^P < 0.01 vs. NC.

We next assessed the effect of PROX1 overexpression on the proliferative, migratory, and invasive abilities of CRC cells. HT-29 cells, which exhibit a low level of PROX1, were employed to perform PROX1 overexpression. The transfection efficiency of the PROX1 overexpression constructs was evaluated using RT-qPCR (Figure 4A). Functional analysis indicated that PROX1 overexpression significantly augmented CRC cell proliferation (Figure 4B) and colony formation (Figure 4C) and flow cytometric analysis showed cell cycle progression to the S phase (Figure 4D), migratory ability (Figure 4E), and invasive properties (Figure 4F) of HT-29 cells. These findings of the functional assay have shown that *in vitro* level PROX1 is involved in CRC cell proliferation, migration, and invasion properties.

### α-SMA-rich CAF potentiates PROX1-expressing CRC cell invasion

**Figure 5:**
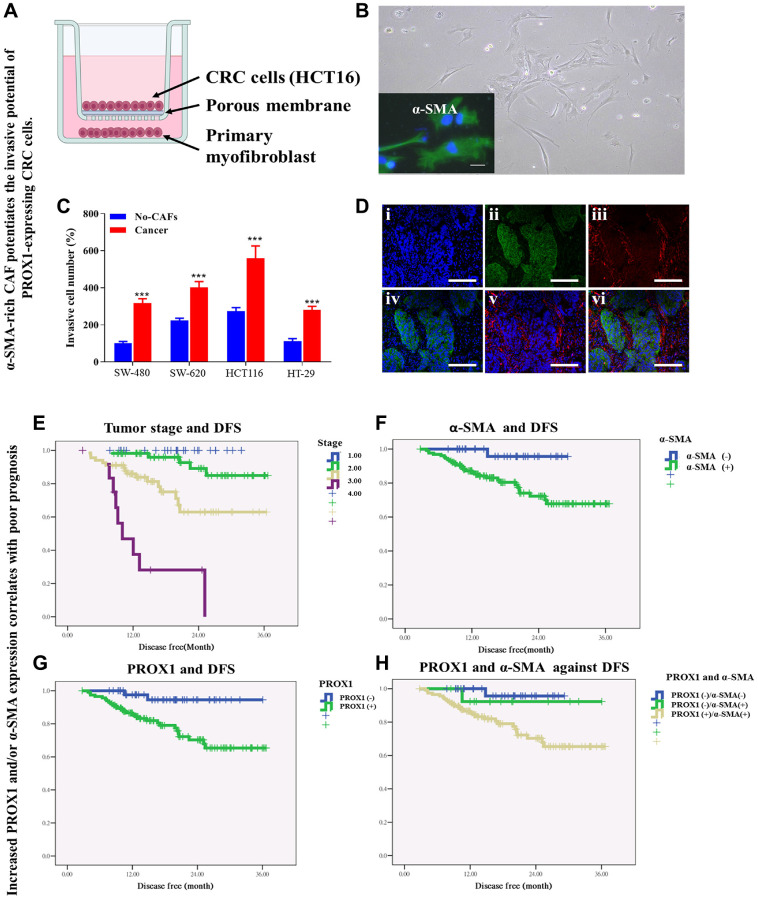
**α-SMA-rich CAF potentiates the invasive potential of PROX1-expressing CRC cells.** (**A**) Schematic representation of α-SMA-rich CAF co-cultured with PROX1-expressing CRC (HCT116) cells. (**B**) Micrograph of α-SMA-rich CAF, with inserted immunofluorescent staining of α-SMA. (**C**) Invasion assay shows SW-480, SW-620, HCT116 and HT-29 cells co-cultured with α-SMA-rich CAF acquired increased invasive potential compared to their counterparts which were not co-cultured with the CAF. (**D**) Immunofluorescence staining of PROX1 and/or α-SMA in CRC tissue sample. Blue – DAPI, Green – PROX1 and Red – α-SMA. Survival rate analysis of colorectal cancer patients. Kaplan-Meier curve analysis shows a correlation between (**E**) tumor stage and disease-free survival, DFS, (**F**) PROX1 expression and DFS, (**G**) α-SMA expression and DFS, and (**H**) co-expression of PROX1 with α-SMA, and DFS.

We assessed the interplay between CAF-associated α-SMA and PROX1, and the impact of these interactions on tumor aggression by coculturing PROX1-expressing CRC cells (SW-480, SW-620, HCT116, and HT-29 cells) with α-SMA-rich CAFs (Figure 5A, 5B). We observed that SW-480, SW-620, HCT116, and HT-29 cells when cocultured with α-SMA-positive CAFs gained greater invasiveness than their control counterparts (Figure 5C). The immunofluorescence staining of the coculture assay revealed that PROX1 and α-SMA were detected simultaneously (Figure 5D). Links were identified between the expression of PROX1/α-SMA and the clinicopathological features of CRC patients. Additionally, an analysis of the clinical significance of PROX1 expression, both individually and in conjunction with α-SMA was conducted comparing CRC with normal colonic mucosa from the same patients (Table 1). Comparison of PROX1 or α-SMA expression in cancerous colon tissue with that in corresponding normal colonic mucosa indicated PROX1 and α-SMA expression were significantly greater in cases with lymph node metastasis (*p*_PROX1_ <0.001, *p*_α-SMA_ <0.001), lymphatic invasion (*p*_PROX1_ <0.002, *p*α_-SMA_ <0.004), and tumor staging (*p*_PROX1_ <0.001, *p*_α-SMA_ <0.001) but non-significant for vascular invasion (*p*_PROX1_ <0.089, *p*_α-SMA_ <0.11; Table 1). Expression of PROX1 or α-SMA was significantly correlated with tumor size and histological type (*p*_PROX1_ <0.001, *p*_-SMA_ <0.001 for both); however, neither PROX1 nor α-SMA expression correlated with patients’ sex or age (Table 1). When taken together as a gene set, PROX1 expression and α-SMA expression had a similar statistical trend. Indeed, CRC cells positive for PROX1 had markedly higher α-SMA expression than those with null PROX1 expression and vice versa (Table 1).

To investigate the association of PROX1 and α-SMA expression with distant metastasis, we used human colon tissue samples and conducted a comparative analysis between CRC cases with lymphatic invasion or lymph node metastasis and those with a localized tumor. We observed significantly higher PROX1 and α-SMA expression in patients of CRC with lymphatic invasion or lymph node metastasis compared with those of CRC without lymphatic invasion and lymph node metastasis (Table 2).

**Table 2:** Correlation between gene-set PROX1 and α-SMA expression and patients' clinicopathological characteristics.

	**PROX1 (+) α-SMA (+)4**	**PROX1 (+) α-SMA (−)3**	**PROX1 (−) α-SMA (−)2**	**PROX1 (−) α-SMA (−)1**	***P*-value**
Patient number (n)	116	0	14	34	
Serosal invasion					<0.001
Positive	111		12	8	
Negative	5		2	26	
Lymphatic invasion					0.006
Positive	65		5	9	
Negative	51		9	25	
Venous invasion					0.217
Positive	53		5	10	
Negative	63		9	24	
Lymph node metastasis					<0.001
Positive	69		7	2	
Negative	47		7	32	
Distant metastasis					0.054
Positive	13		0	0	
Negative	113		14	34	
Stage					<0.001
I	0		2	25	
II	45		5	7	
III	58		7	2	
IV	13		0	0	

This was following the IHC data, where α-SMA expression in cancerous tissue was markedly higher than that in paired normal tissue, with α-SMA-positive PROX1-expressing cancer cells exhibiting significantly higher invasive potential than those with null α-SMA expression. All these results indicated a close connection or crosstalk between PROX1 and α-SMA expression and their potential modulatory role in invasion or metastasis in CRC cells. Notably, an increase in the PROX1 and α-SMA expression correlates with poor prognosis. Survival analyses showed shorter disease-free survival (DFS) in patients with late-stage CRC (Figure 5E) and considerably shorter DFS in patients with PROX1+ CRC than in those with PROX1- CRC (Figure 5F). A similar trend was observed for α-SMA expression and DFS (Figure 5G). Collectively and with results for individual expression, these results indicate that increased PROX1 and α-SMA phrases are negatively correlated with DFS (Figure 5H).

### PROX1 knockdown prevents CAF transformation in the CRC TME

**Figure 6:**
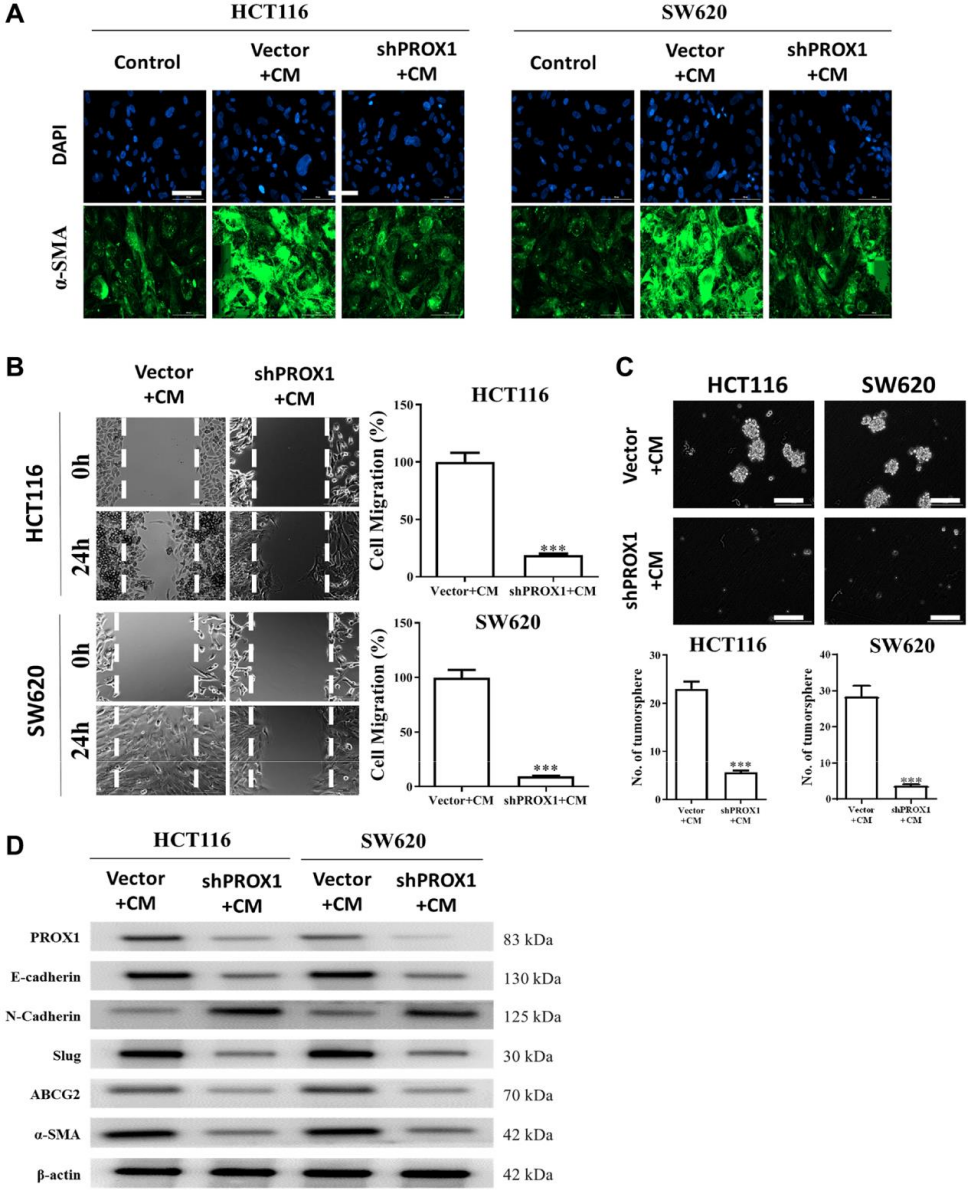
**PROX1 inhibition prevented cancer-associated fibroblast (CAF) transformation.** (**A**) Representative immunofluorescence images of CAFs transformed by PROX1 inhibition in HCT116 and SW620 cells. Reduced expression of alpha-smooth muscle actin (α-SMA) was observed. PROX1-inhibited cells in the presence of condition medium (CM) showed reduced migratory (**B**) and tumor sphere-generating abilities (**C**). (**D**) Western blot analysis demonstrated the expressions of the oncogenic markers, stemness markers, CAFs markers (α-SMA), and PROX1, in PROX1-inhibited and control samples under the influence of CM. ^*^p < 0.05, ^**^p < 0.01, ^***^p < 0.001.

Inside TME, CAFs are a key facilitator of colon cancer progression [31]. CRC cell lines were cocultured with conditioned medium (CM) from CAFs or NFs. We looked at whether reduced PROX1 expression could prevent CAF transformation and observed that PROX1-knockdown in SW620 and HCT116 cells resulted in normal fibroblasts having a significantly lower ability to transform into CAFs compared with those in the untreated counterpart cells (Figure 6A). More to the point, CAFs with PROX1-knockdown demonstrated significantly lower wound-healing ability—that is, less migration (Figure 6B) and tumor-sphere generation (Figure 6C). These observations were backed by Western blotting analysis, which showed the increased expression of the oncogenic markers, stemness markers, CAF markers (α-SMA), and PROX1 and increased expressions of ABCG2 in CAF-resistant cells; however, all these markers were significantly suppressed after PROX1 knockdown (Figure 6D).

### Effect of PROX1 knockdown on tumor growth *in vivo*

**Figure 7:**
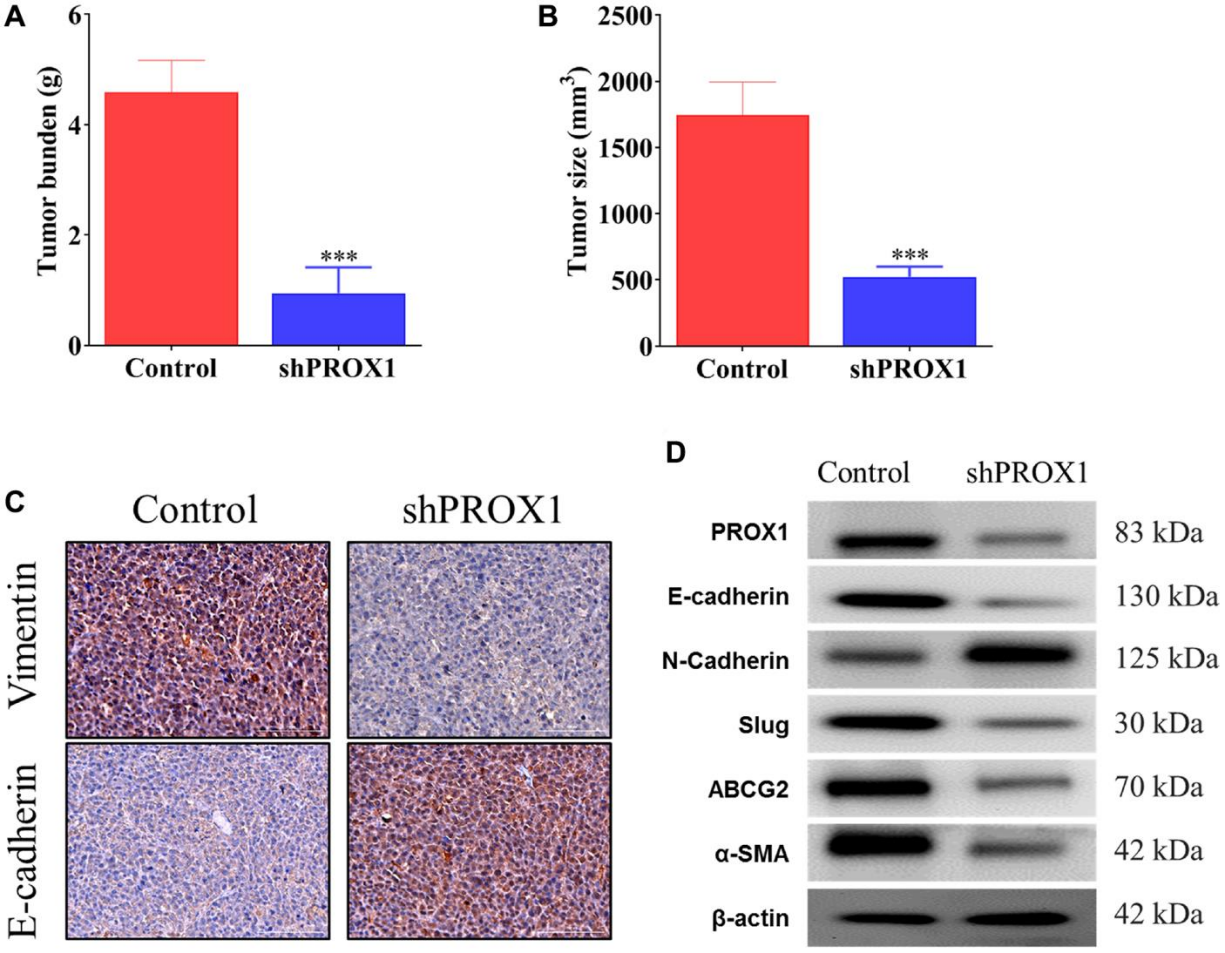
**PROX1 knockdown inhibits xenograft tumor growth in nude mice.** PROX1 knockdown reduced HCT116 cell-derived xenograft tumor growth in nude mice (*n* = 5). (**A**) Statistical comparison of differences in tumor weights and (**B**) Tumor size between the sh-NC (Control) and PROX1-shRNA#1 group. Growth curve of HCT116 xenograft tumors monitored in the sh-NC and PROX1-shRNA#1 group. (**C**) Immunohistochemistry analysis of epithelial-mesenchymal transition (EMT) marker expression (magnification, ×400; scale bar, 50 μm) in xenograft tumor tissues in the sh-NC and shRNA-PROX1#1 group. (**D**) Western blot analysis of PROX1 expression in tumor tissue samples together with other key markers associated with the EMT process and resistance. β-actin is the loading control. The data are presented as the mean ± standard deviation. ^*^P < 0.05 and ^**^P < 0.01 vs. sh-NC.

The role of PROX1 in tumorigenicity was further evaluated *in vivo*. For this, HCT116 cells were stably transfected with an empty shRNA vector and PROX1-shRNA#1 with the CM, as described earlier. These PROX1-altered HCT116 cells were subcutaneously injected into the backs of the nude mice. Notably, in xenografts, as presented in Figure 7A, the tumors developed from the cells transfected with PROX1-shRNA#1 were significantly smaller than the cells transfected with empty shRNA vector control. Significantly lower tumor weight and volume were discovered in the PROX1-shRNA#1 group compared with the control groups (Figure 7B). Additionally, the expression of EMT markers was analyzed using IHC of tumor tissue sections, and the PROX1 knockdown tumors were discovered to exhibit lower expression of vimentin and induced expression of E-cadherin (Figure 7C). Furthermore, Western blotting confirmed lower expression of oncogenic markers, stemness markers, CAF markers (α-SMA), and PROX1 as well as lower expression of ABCG2 in the PROX1-knockdown tumor samples as compared with vector control (Figure 7D).

## DISCUSSION

The Tumor Microenvironment (TME) plays a pivotal role in shaping the behavior of various malignancies through intricate molecular interactions [32]. Within the TME, α-SMA-rich Cancer-Associated Fibroblasts (CAFs) are active in governing processes central to cancer growth and metastasis [8]. Lacina et al. highlighted that CAFs expressing α-SMA have a distinct gene expression profile compared to regular fibroblasts, and they can induce oncogenic or Cancer Stem Cells (CSCs) characteristics in normal epithelial cells [9]. The homeobox gene PROX1, a conserved transcription factor, is pivotal in determining cell destiny. Its overexpression in numerous cancers often signifies a more aggressive disease course [33–35]. Specifically, in Colorectal Cancer (CRC), PROX1 is critical for instilling and preserving stem-cell-like attributes [36–39]. Given the high recurrence rate of CRC post-surgery and the existing knowledge gaps about recurrence mechanisms, our study aimed to elucidate the relationship between elevated PROX1 and α-SMA expression, cancer progression, clinical outcomes, and their influence on CAFs in the CRC TME. The TME’s influence on cancer cell behavior and differentiation is well-documented, with the dynamics within the TME and CSCs activity being central to tumor progression [7, 40].

The result indicated PROX1 was overexpressed in tumor tissue compared with its normal counterpart; furthermore, loss or gain of PROX1 attenuated cell proliferation, migration, and invasion, both *in vivo* and *in vitro*. Flow cytometric analysis revealed that PROX1 knockdown induced G1 phase arrest, whereas PROX1 overexpression promoted the G1–S phase transition. IHC and immunofluorescence analyses revealed that PROX1 and α-SMA were significantly overexpressed in the TME. Notably, PROX1 and α-SMA were found to play crucial roles in the acquisition of invasiveness, facilitation of disease progression, and prediction of clinical outcomes. PROX1 and α-SMA overexpression were observed in CRC clinical samples. IHC staining indicated significant expression of α-SMA in the tumor matrix around cancer cells. IHC staining revealed a strong correlation of PROX1 with α-SMA in CRC. Our findings indicated that higher expression of PROX1 and α-SMA in CRC tissue was positively correlated with tumor cell invasiveness and advanced TNM stage. Furthermore, as confirmed in Table 2, our results demonstrated a strong association between PROX1 and α-SMA expression and distant metastasis. The results confirmed significantly higher PROX1 and α-SMA gene expression in patients with CRC with lymphatic invasion or lymph node metastasis compared with those without.

Furthermore, advanced CRC cases exhibited significantly higher PROX1 and α-SMA expression than early-stage tumors. A direct relationship was observed between tumor size and the expression of these genes, suggesting their intertwined roles and their collective influence on CRC’s malignant potential. The analysis of Disease-Free Survival (DFS) indicated that higher levels of PROX1 and α-SMA were correlated with increased recurrence rates, highlighting their potential as prognostic markers and might be key therapeutic targets for CRC.

The study suggests that a dual targeting approach, focusing on both PROX1 and α-SMA, might yield better outcomes. The hypothesis is based on several observations: the association of α-SMA-rich CAFs with unfavorable clinical outcomes in various cancers [41], the importance of PROX1 in cell survival, proliferation, and its link with the intestinal stem-cell-like phenotype [42], and the observed over-expression of both genes in the colonic tumor stroma. Additionally, the upregulation of their downstream targets, Snail and fibronectin, in the CRC stroma, further emphasizes their significance in disease progression and prognosis [43]. To our understanding, this study is pioneering in showcasing the relationship between PROX1 and α-SMA expression and their collective role in CRC progression by modulating the CRC TME. We also highlight their potential as indicators of unfavorable clinical outcomes.

Our study elucidates the influential roles of PROX1 and α-SMA in the CRC tumor microenvironment, highlighting their potential as critical prognostic biomarkers and therapeutic targets. Despite these insights, several limitations require consideration for a more accurate interpretation of our findings. Firstly, the reliance on the TCGA data acquisition confines our analysis to existing datasets, introducing potential biases, and questioning the generalizability of our findings. Secondly, our study’s focus predominantly on PROX1 and α-SMA may overlook other significant molecular players and pathways involved in CRC progression. Thirdly, despite the promising prospect of developing inhibitors targeting the PROX1/α-SMA gene set, this approach remains exploratory and requires further comprehensive future research and validation.

## CONCLUSION

**Figure 8:**
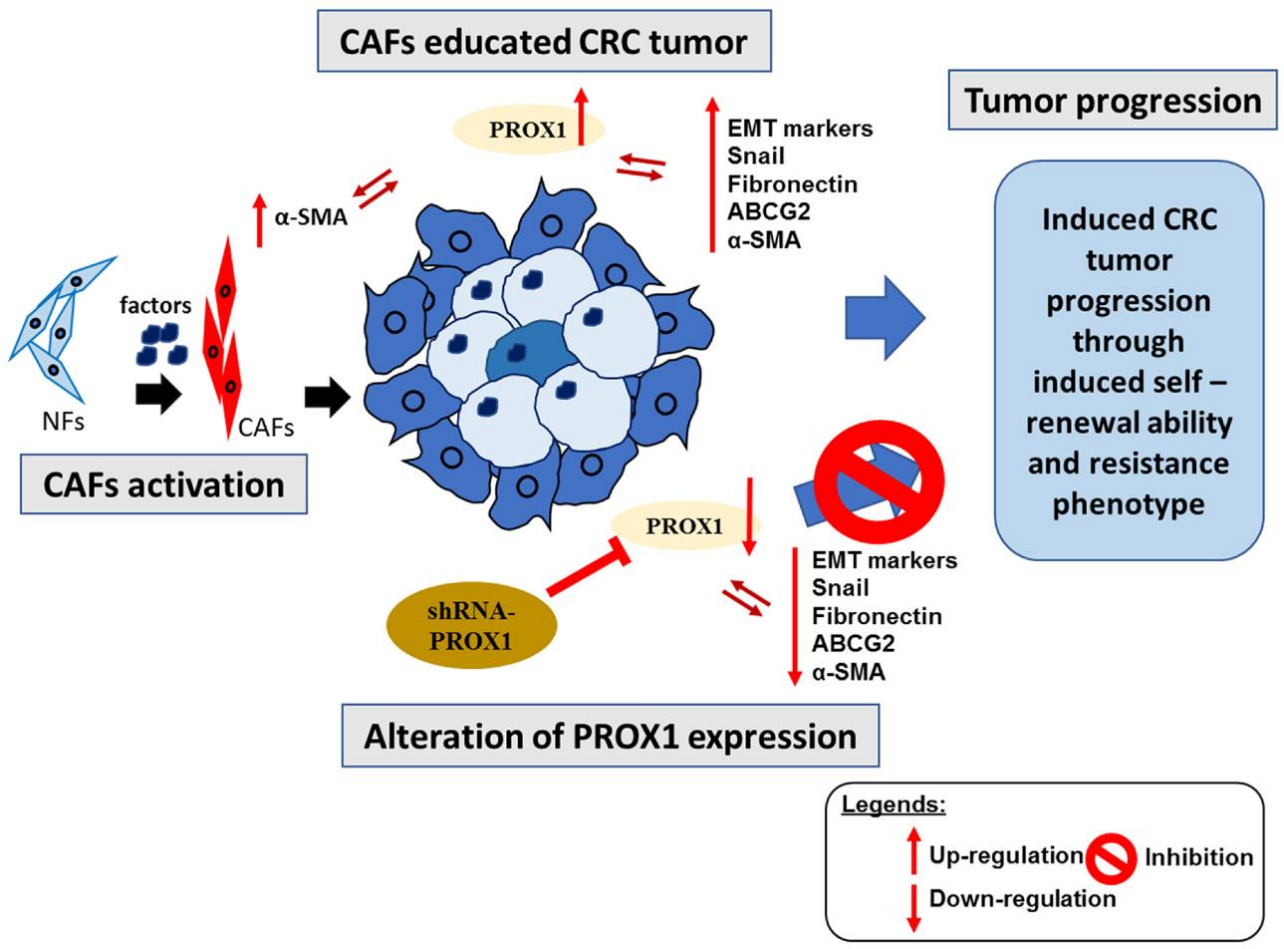
**Overall summary of our study.** CAFs educated CRC cells under the modulation of the expression of PROXA1 results in induced invasive, tumor progression and resistance properties. Furthermore, the reversal of aforementioned properties was observed after the shRNA-PROXA1 mediated inhibition of PROXA1, resulting in resensitization of CRC tumor towards the therapy.

The findings validate the combined PROX1/α-SMA gene set as a prospective prognostic biomarker and a central regulator in CRC progression and its TME. Strategically targeting this gene combination offers a promising avenue for innovative therapeutic strategies, not only enhancing CRC treatment efficacy, but also potentially mitigating tumor progression, metastasis, and recurrence by modulating the EMT and CAF dynamics within the TME, as depicted in Figure 8. As we advance in this field, the development and clinical validation of small-molecule inhibitors targeting PROX1/α-SMA become imperative, paving the way to refine and optimize CRC therapeutic interventions.
